# Spatial memory is impaired by peripubertal GnRH agonist treatment and testosterone replacement in sheep

**DOI:** 10.1016/j.psyneuen.2016.10.016

**Published:** 2017-01

**Authors:** D. Hough, M. Bellingham, I.R.H. Haraldsen, M. McLaughlin, M. Rennie, J.E. Robinson, A.K. Solbakk, N.P. Evans

**Affiliations:** a Institute of Biodiversity Animal Health and Comparative Medicine, College of Medical Veterinary and Life Sciences, University of Glasgow, Glasgow, G61 1QH, UK; b Department of Medical Neurobiology, Division of Clinical Neuroscience, Oslo University Hospital — Rikshospitalet, 0027, Oslo, Norway; c Division of Veterinary Bioscience and Education, School of Veterinary Medicine, College of Medical Veterinary and Life Sciences, University of Glasgow, Glasgow, G61 1QH, UK; d Department of Psychology, University of Oslo, Pb 1094 Blindern, 0317 Oslo, Norway; e Department of Neuropsychology, Helgeland Hospital, Mosjøen, Norway

**Keywords:** Spatial orientation, Spatial memory, Hippocampus, GnRH, Puberty, Gender dysphoria

## Abstract

Chronic gonadotropin-releasing hormone agonist (GnRHa) is used therapeutically to block activity within the reproductive axis through down-regulation of GnRH receptors within the pituitary gland. GnRH receptors are also expressed in non-reproductive tissues, including areas of the brain such as the hippocampus and amygdala. The impact of long-term GnRHa-treatment on hippocampus-dependent cognitive functions, such as spatial orientation, learning and memory, is not well studied, particularly when treatment encompasses a critical window of development such as puberty. The current study used an ovine model to assess spatial maze performance and memory of rams that were untreated (Controls), had both GnRH and testosterone signaling blocked (GnRHa-treated), or specifically had GnRH signaling blocked (GnRHa-treated with testosterone replacement) during the peripubertal period (8, 27 and 41 weeks of age). The results demonstrate that emotional reactivity during spatial tasks was compromised by the blockade of gonadal steroid signaling, as seen by the restorative effects of testosterone replacement, while traverse times remained unchanged during assessment of spatial orientation and learning. The blockade of GnRH signaling alone was associated with impaired retention of long-term spatial memory and this effect was not restored with the replacement of testosterone signaling. These results indicate that GnRH signaling is involved in the retention and recollection of spatial information, potentially via alterations to spatial reference memory, and that therapeutic medical treatments using chronic GnRHa may have effects on this aspect of cognitive function.

## Highlights

• Peripubertal GnRHa impaired long-term spatial reference memory.
• This GnRHa-effect was not counteracted with testosterone replacement in rams.
• Traverse times of spatial orientation and learning tasks were unaffected by GnRHa.
• GnRHa exaggerated emotional reactivity during these spatial tasks.
• Testosterone replacement decreased emotional reactivity and motivation in tasks.

## 1 Introduction

Gonadotropin-releasing hormone (GnRH) is a hypothalamic decapeptide that binds to GnRH receptors (GnRHR) in the anterior pituitary gland to stimulate the release of the gonadotropins; luteinizing hormone (LH) and follicle stimulating hormone (FSH). While GnRH neuronal cell bodies are principally located in the preoptic area of the hypothalamus and the majority of their axons project to the median eminence, some GnRH axons extend to other regions of the central nervous system (CNS), including the limbic system (Silverman et al., 1987). GnRH can cross the blood-brain barrier, from the median eminence, into the third ventricle cerebrospinal fluid (Caraty and Skinner, 2008), so GnRH could have effects on brain function. Caraty and Skinner (2008) reported that only extremely high intravenous doses of exogenous GnRH (2.5 μg and 1 mg injection into the jugular vein of ewes) resulted in elevated GnRH in the third ventricle to a physiologically relevant level. Chronic GnRH agonist (GnRHa) treatment leads to GnRHR desensitization via receptor-G protein uncoupling, internalization and recycling of GnRHR in the pituitary gland (Ferguson et al., 1996, Armstrong et al., 2011), which suppresses activity within the hypothalamic-pituitary-gonadal (HPG) axis, including gonadotropin and gonadal steroid signaling. Long-term GnRHa-treatment is therefore used in pediatric human medicine as a co-treatment for conditions such as early onset gender dysphoria, central precocious puberty, idiopathic short stature, growth hormone deficiency, congenital adrenal hyperplasia, and severe hypothyroidism (Carel et al., 2009, Hembree et al., 2009). GnRHR expression and GnRH binding are present in extra-pituitary tissues (Hapgood et al., 2005), including brain regions such as the hippocampus and other limbic structures (Jennes et al., 1997, Albertson et al., 2009, Skinner et al., 2009, Schang et al., 2010). Therefore, long-term GnRHa-treatment may have additional cognitive and behavioral effects, due to a blockade of GnRH signaling outside the HPG axis (Carel et al., 2009, Hembree et al., 2009). Indeed, a growing body of evidence supports numerous and diverse autocrine, paracrine and endocrine roles for GnRH/GnRHa and GnRHR outside the pituitary gland (Hsueh and Schaeffer, 1985, Hapgood et al., 2005, Prange-Kiel et al., 2008).

Peripubertal GnRHa-treatment raises additional concerns, as this is a critical window for neuronal development and programming (Berenbaum and Beltz, 2011). In humans, adolescence is associated with marked changes in behavior and cognition associated with neurocognitive maturation and/or changes in pubertal hormones (Ernst et al., 2009). Adolescents typically demonstrate increased risk-taking, exploratory and obsessive behaviors (Ernst et al., 2009) and emotional reactivity, e.g. heightened anxiety (reviewed in Buchanan et al., 1992) and responsiveness to stressful situations (Walker et al., 1995, Hascoet et al., 1999). Adolescence is also a time when many neuropsychiatric disorders, as well as behavioral and emotional problems, become apparent (Eaton et al., 2008, Casey and Jones, 2010). Whether developmental changes in cognition and behavior are directly affected by patterns of GnRH secretion seen at this time, is not known.

Previous studies with an ovine model have demonstrated that pharmacological blockade of the pubertal transition with a GnRHa (goserelin acetate) results in sex specific changes in cognition and behavior. GnRHa-treated males were more likely to display risk-taking behavior in a food acquisition task (Wojniusz et al., 2011), and heightened emotional reactivity (i.e. emotional and behavioral responses to a fearful situation) in early adulthood, but decreased emotional reactivity at a later age (Evans et al., 2012). Furthermore, peripubertal GnRHa-treatment is accompanied by changes in amygdala volume (Nuruddin et al., 2013a) and hippocampal gene expression (Nuruddin et al., 2013b). In the latter study, changes were observed in mRNA expression for genes relating to endocrine signaling and synaptic plasticity, but these were not accompanied by significant differences in spatial orientation (Wojniusz et al., 2013), which is a hippocampus-dependent function (Burgess et al., 2002). A tendency was noted, however, for GnRHa-treated animals of both sexes to traverse a spatial maze slower than untreated controls (Nuruddin et al., 2013b, Wojniusz et al., 2013). It is possible that the ability to measure such effects on spatial orientation were limited, in that study, by the use of too simplistic a maze design and an associated ceiling effect. Furthermore, Robinson et al. (2014) noted that the interpretation of behavioral data (e.g. novelty-seeking) in this ovine model is complex, because the assessed behaviors may be pre- and perinatally programmed and/or strongly influenced by the emotional reactivity of the animal as it adapts to the environment in which it finds itself.

In this study, the aim is to investigate the risks involved with peripubertal GnRHa-treatment for the development of spatial orientation, learning and memory. We focused on the male and extend our initial results, by investigating the effects of chronic peripubertal GnRHa-treatment on age-related changes in spatial orientation in a more complex maze design, while also taking into account any associated changes in emotional reactivity. Lee et al. (2006) demonstrated that sheep are capable of retaining spatial information, or solving strategies, for at least 6 weeks. Spatial memory is also sexually differentiated (Jonasson, 2005) and influenced by testosterone (Celec et al., 2015). Thus, in this study we tested the hypothesis that chronic peripubertal GnRHa-treatment would affect long-term spatial memory, and we attempted to dissociate between two components of long-term spatial memory, namely spatial reference memory (i.e. recollection of familiar spatial cues) and spatial working memory (i.e. solving strategy based on sequence of spatial cues). Finally, we included an additional group that received testosterone replacement, in conjunction with GnRHa-treatment, to allow differentiation between the effects of blocking GnRH and/or gonadal steroid signaling.

## 2 Materials and methods

### 2.1 Animals and treatment

This study was conducted at the University of Glasgow Cochno Farm and Research Centre (55° 55′N) in accordance with Home Office Regulations (Project License: 60/4422). Scottish Mule Texel crosses born between 23 March and 12 April 2013 were kept with dams until weaning at 21 weeks of age. Sheep were grazed on pasture, except during lambing and behavioral trials, when they were housed indoor with ad libitum access to hay or silage, and supplements (i.e. protein or mineral concentrates) according to standard management practices. Male lambs from single sex litters were used to rule out potential effects of the prenatal steroid environment and twins or triplets were randomly assigned to different treatment groups to minimize maternal effects. Treatment groups consisted of: 1) untreated (Control); 2) GnRHa-treated (GnRHa); and 3) GnRHa-treated rams that also received testosterone replacement (GnRHa+T). GnRHa-treatment consisted of a subcutaneous implant of goserelin acetate (Zoladex 3.6 mg, kindly donated by Astra Zeneca, Macclesfield, UK) every 4 weeks, from 8 to 44 weeks of age, as the average age of pubertal onset in male sheep is 10 weeks of age (Wood and Foster, 1998). Testosterone replacement consisted of intramuscular injections of testosterone cypionate (A6960-000, Steraloids, Newport, USA) dissolved in vegetable oil at doses estimated to replicate endogenous profiles in controls (assumed to be as reported by Robinson et al., 2014). Testosterone cypionate was administered once every 2 weeks as follows: 16 weeks of age, 50 mg/mL; 18–24 weeks of age, 120 mg/mL; 26–30 weeks of age, 160 mg/mL; 32–44 weeks of age, 240 mg/mL; and 46 weeks of age, 136.4 mg/mL. Initially, 139 rams were assigned to the project, namely Control *n* = 60, GnRHa *n* = 55 and GnRHa+T *n* = 24, of which 12 (Control *n* = 4, GnRHa, *n* = 6, Testo *n* = 2) were removed from the study due to illness. There were twice as many Control and GnRHa rams as Testo rams, because half of the Control and GnRHa groups continued into another study.

### 2.2 Assessment of spatial orientation and learning

#### 2.2.1 Spatial maze design

**Fig. 1:**
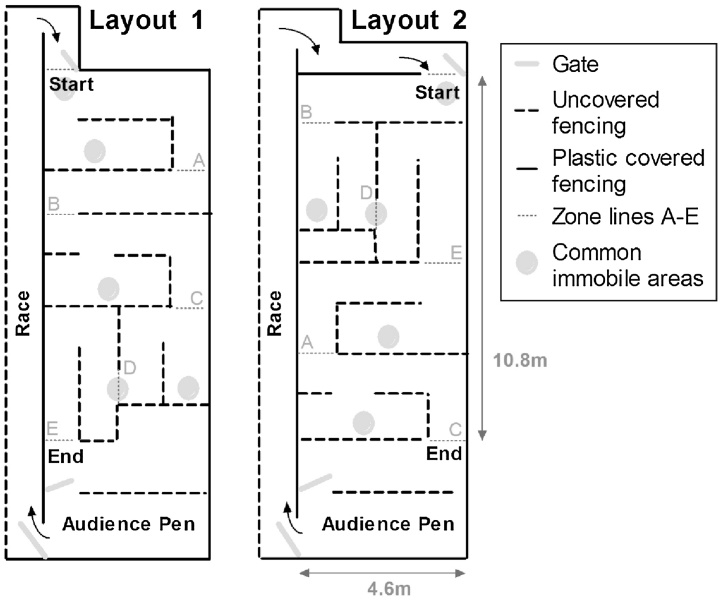
Spatial maze layouts that were used for the assessment of spatial orientation, learning and long-term spatial memory (Layout 1), as well as the novel maze (Layout 2) in which traps within the same-lettered zones were rearranged.

The spatial maze was a modification of the design described by Wojniusz et al. (2013) and Lee et al. (2006) in that the complexity of the maze was increased by inclusion of ‘traps’ that forced animals to move away from their conspecifics in the audience pen, to progress through the maze (Fig. 1, layout 1). The maze was constructed in the same barn where sheep were housed. The maze was bordered by metal fencing covered with black plastic sheeting, to obstruct the view of activity outside the maze area. The internal walls of the maze remained uncovered, thus the audience pen was visible at all times to utilize the flocking instinct as motivation to move through the maze and reunite with their mothers (8 weeks of age) and flock members (8, 27 and 41 weeks of age). On each test day, approximately 30 randomly selected sheep were kept in the audience pen with ad libitum access to hay and water.

#### 2.2.2 Assessment of spatial orientation and learning

Changes in **spatial orientation** were assessed as the performance of sheep in the first maze attempt of the day, at each age. **Spatial learning** was assessed as the performance of sheep over three separate maze attempts within the same day (each attempt separated by ∼2 h). During an attempt, each sheep was calmly ushered from the audience pen to the start of the maze. Of the rams that did not complete the maze within the 5 min time limit, some remained near the entrance throughout, whereas others traversed some of the maze before returning to the entrance in an attempt to exit the maze via their point of entry. It was observed that some sheep paused in certain areas within the maze, as indicated by grey circles in Fig. 1. Sheep failing to complete the maze within 5 min were ushered back to the audience pen via the maze entrance, so that the correct route remained unknown. On the last attempt of the day, unsuccessful sheep proceeded to the audience pen via the quickest route.

#### 2.2.3 Recorded observations

Spatial performance was individually assessed at 8 weeks (pre-pubertal, prior to hormone treatment), 27 weeks (post-pubertal for Controls, during the breeding season, 19 weeks after the start of hormone treatment), and 41 weeks of age (post-pubertal, during non-breeding season, 33 weeks after the start of hormone treatment). **Traverse time** (min:s:ms) was recorded as the time taken to move from the entrance to finish line (line E in layout 1 of Fig. 1); judged on the placement of the front legs across the line (5 min = incomplete). **Progress through the maze** was recorded as the time difference moving between lines A to E. **Emotional reactivity** was recorded as the number of vocalizations, escape attempts, urinations and defecations. An escape attempt was defined as any proactive effort to move through, over or under a wall.

### 2.3 Assessment of long-term spatial memory

#### 2.3.1 Training

Long-term spatial memory training was done, with a group of randomly selected animals (Control *n* = 19, GnRHa *n* = 22, GnRHa + T *n* = 22) over two days, shortly after the 41-week spatial orientation and learning assessment, using the same maze layout (Fig. 1, Layout 1). Training was a two-stage process, which consisted of education and confirmation runs. For education runs, each sheep was given 10 consecutive attempts to complete the maze within 1 min. After each unsuccessful attempt, the ram was calmly escorted through the remainder of the maze, via the finish line, to the audience pen. Sheep completed the education run when they were able to traverse the maze within 1 min on two successive attempts. When all animals had completed the education run, they underwent a confirmation run, which consisted of two attempts to complete the maze within 1 min. If successful, training was completed. If unsuccessful, the sheep had a further 8 attempts to complete the maze within 1 min, i.e. a second set of education runs, followed by two further confirmation runs. A maximum of 3 sets of education runs were conducted within the same day. The total number of attempts during education and confirmation runs was recorded for each ram, together with the quickest traverse time, to serve as a measure of the ease of training.

#### 2.3.2 Assessment of long-term memory

Retention of long-term spatial memory was assessed 4 weeks after training was completed (45 weeks of age). Each sheep was given one maze attempt (Fig. 1, Layout 1) with traverse times (incomplete = 5 min) and progress through maze zones recorded.

#### 2.3.3 Assessment of familiarity in a novel maze design

Immediately after assessment of long-term memory, each sheep was given one attempt to traverse a new spatial maze layout, which contained the same ‘traps’ but in a different order or orientation (Fig. 1, Layout 2). Maze traverse times (incomplete = 5 min) and progress through maze zones were recorded.

### 2.4 Statistical analysis

**Fig. 2:**
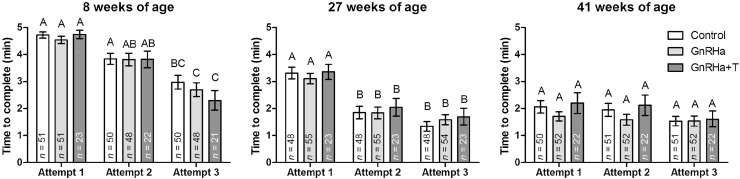
Spatial orientation (Attempt 1 only) and spatial learning (Attempts 1–3) performance was measured by the mean ± s.e.m. maze traverse times of rams as they aged. At 8 weeks of age, all rams remained untreated, whereas at 27 and 41 weeks of age, GnRHa-treatment had been administered for 19 and 33 weeks respectively. Different letters on top of bars indicate significant differences between treatment group means at that particular age, as determined by a Tukey post hoc test from a two-way ANOVA (Treatment × Attempt). Control: untreated rams; GnRHa: GnRHa-treated rams; GnRHa+T: GnRHa-treated rams that also received testosterone replacement.

For each animal, the emotional reactivity parameters were normalized by expression relative to the time spent in the maze. The proportion of time spent in each zone was calculated as a percentage of total time spent in the maze. Data were excluded from analysis where performance was judged to have been compromised because of temporary incapacity, i.e. health concerns. In addition, data were excluded from analysis where animals escaped from the maze area or jumped over internal maze walls. Exclusion of data was done by specifying a missing value for the relevant response variable(s) in that particular maze attempt (*n* specified in Fig. 2). Urination and defecation frequencies were too low for statistical analyses to yield meaningful results.

All statistical analyses were performed with R software (Version 3.2.1, © 2015 The R Foundation for Statistical Computing Platform) using the RStudio interface (Version 0.99.467, © 2009–2015 RStudio Inc.). Where data were not normally distributed, the response variable was log-transformed. Response variables were analyzed using the generalized linear model (GLM) function using a Gaussian distribution; ram identity was included as an explanatory variable to account for individual variation across time or respective maze attempts. Effects of age and treatment on **spatial orientation** were assessed with data from the first attempt of the maze, across all ages, using a two-way ANOVA (Treatment × Age). Effects of treatment on **spatial learning**, over three consecutive maze attempts, were assessed with two-way ANOVA (Treatment × Maze attempt) at each respective age. One-way ANOVA was used to assess the effects of treatment on the ease of maze training (number of training attempts), as well as traverse times upon completion of training. Effects of treatment on **long-term spatial memory** were tested by comparison of traverse times at: 1) 45 weeks of age only (one-way ANOVA); 2) 41 (last training attempt) versus 45 (the assessment attempt) weeks of age (two-way ANOVA: Treatment × Time). The effect of maze design familiarity was examined by comparison of the traverse time of maze layouts 1 and 2 (two-way ANOVA: Treatment × Maze Layout). Tukey Honest Significant Difference (TukeyHSD) post hoc test was used to assess where significant differences existed between treatment groups. All graphs represent means and standard errors of the mean. Statistical *P*-values <0.05 were considered significant.

## 3 Results

### 3.1 Spatial orientation **-***Completion of maze at the first attempt*

#### 3.1.1 Traverse time

The mean traverse time decreased significantly (*P* < 0.001) with age (Fig. 2 & Table 1) and was accompanied by a progressive increase in the proportion of rams that successfully completed the maze (23, 71, 85% at 8, 27 and 41 weeks, respectively). There were no significant effects of either the GnRHa or GnRHa + T treatments on the time taken to complete the maze at the first attempt, at either 27 or 41 weeks of age (Fig. 2 & Table 1).

#### 3.1.2 Progress through maze zones

**Fig. 3:**
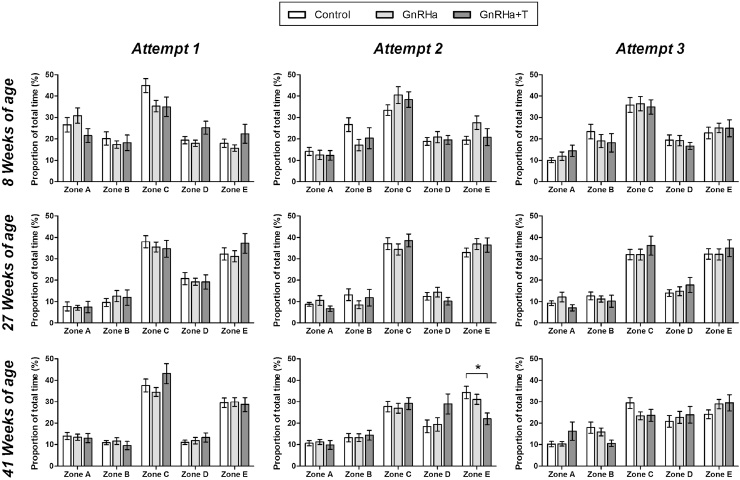
Proportionate time spent in each zone of the maze during spatial orientation (Attempt 1 only) and spatial learning (Attempts 1–3) assessments as rams aged. At 8 weeks of age, all rams remained untreated, whereas at 27 and 41 weeks of age, GnRHa-treatment had been administered for 19 and 33 weeks respectively. Control: untreated rams; GnRHa: GnRHa-treated rams; GnRHa+T: GnRHa-treated rams that also received testosterone replacement. *P < 0.05.

The proportion of time spent in the different maze zones varied significantly as a function of age (Fig. 3, Attempt 1 & Table 1). Specifically, while rams spent the greatest proportion of time in maze zone C, regardless of age, the proportion of time spent in zones A, B and D decreased, and zone E increased significantly (*P* < 0.05) with age. Typically, the largest changes were seen between 8 and 27 weeks of age. There were no significant effects of treatment on the proportion of time spent in the different maze zones.

#### 3.1.3 Emotional reactivity

**Fig. 4:**
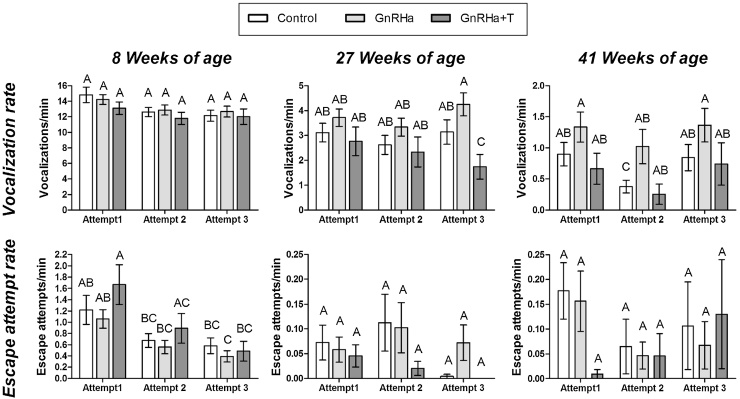
Measures of emotional reactivity, expressed as the mean ± s.e.m. rates of vocalizations and escape attempts, during spatial orientation (Attempt 1 only) and spatial learning (Attempts 1–3) assessments as rams aged. At 8 weeks of age, all rams remained untreated, whereas at 27 and 41 weeks of age, GnRHa-treatment had been administered for 19 and 33 weeks respectively. Different letters on top of bars indicate significant differences between treatment group means, as determined by a Tukey post hoc test from a two-way ANOVA (Treatment × Attempt) at that particular age. Control: untreated rams; GnRHa: GnRHa-treated rams; GnRHa+T: GnRHa-treated rams that also received testosterone replacement.

Emotional reactivity improved with age, as seen by the progressive decrease in vocalization rate and escape attempt frequency. The overall mean vocalization rate decreased significantly (*P* < 0.001) (Fig. 4 & Table 1) from 14.3 ± 0.50 vocalizations/min at 8 weeks, to 3.3 ± 0.23 and 1.0 ± 0.13 at 27 and 41 weeks of age, respectively. There was no significant effect of treatment on vocalization rate, regardless of age (Table 1). Escape attempt frequency also decreased significantly (*P* < 0.001) with age (Fig. 4 & Table 1), from an overall average of 1.23 ± 0.14 attempts/min at 8 weeks, to only 0.06 ± 0.018 and 0.14 ± 0.035 attempts/min at 27 and 41weeks of age, respectively. There was a significant (*P* < 0.05) Treatment × Age interaction, as escape attempt frequency decreased to a greater extent in GnRHa + T compared to Control and GnRHa animals, as they aged (Fig. 4, Attempt 1).

### 3.2 Spatial learning **-***Completion of maze with same-day repeated attempts*

#### 3.2.1 Traverse time

The mean traverse times, across the three maze attempts at each age, are shown in Fig. 2 with the associated two-way ANOVA *P*-value summary in Table 2. At 8 weeks of age, there was a significant (*P* < 0.001) reduction in the average traverse time; 18.2% between attempts 1 and 2, and 30.5% between 2 and 3. At 27 and 41 weeks of age there was also a significant (*P* < 0.001) overall reduction in traverse time across the three attempts; at 27 weeks it was 41.2% between attempts 1 to 2, and 19.5% between 2 and 3, whereas at 41 weeks it was 5.4% between attempts 1 to 2, and 17.1% between 2 and 3.

While there was no overall effect of treatment on the traverse times across the three attempts at 27 and 41 weeks of age, when only the GnRHa and GnRHa+T groups was compared at 41 weeks of age, a trend (*P* = 0.054) was noted for traverse time to be longer in GnRHa+T compared to GnRHa rams; an effect that was most pronounced in attempts 1 and 2.

#### 3.2.2 Progress through maze zones

The mean proportional times spent in the maze zones across all three attempts at 8, 27 and 41 weeks of age, are shown in Fig. 3 with the associated two-way ANOVA *P*-value summary in Table 2. At 8 weeks of age, the proportion of time spent in zone A significantly (*P* < 0.001) decreased and Zone E significantly (*P* < 0.05) increased, across the three maze attempts. At 27 weeks of age, although the pattern of zone usage, across the three maze attempts, was more consistent compared to 8 weeks of age, significant (*P* < 0.001) changes in the proportion of time spent in each maze zone were observed. Specifically, animals spent increasingly more (*P* < 0.001) time in zone A, less time in zone C (*P* < 0.005), whereas animals only spent less time (*P* < 0.05) in zone B during attempt 2. At 41 weeks of age, while the proportion of time spent in the individual maze zones was dominated by zone C and E during attempt 1, it became more equally spread across zones during attempts 2 and 3. This resulted in an overall significant (*P* < 0.001) effect of maze attempt, with a significant increase in the proportion of time spent in zones B (*P* < 0.01) and D (*P* < 0.001), and a significant (*P* < 0.001) decrease in the proportion of time spent in zone C.

The only statistically significant (*P* < 0.05) effect of treatment on the proportional times spent in any of the maze zones, at either 27 or 41 weeks of age, was in zone D. This was because, at 41 weeks of age, the GnRHa+T group spent a higher proportion of time in zone D than the Control and GnRHa groups, particularly during maze attempt 2. At 27 weeks of age, the GnRHa animals tended to spend the greatest, and the GnRHa+T animals the least, proportion of time in zone A (statistical comparison of GnRHa and GnRHa+T groups only: Treatment *P* = 0.081, Attempt *P* < 0.001, Treatment × Attempt *P* = 0.578).

#### 3.2.3 Emotional reactivity

Mean vocalization and escape attempt rates across all three attempts at each age are shown in Fig. 4 with the associated two-way ANOVA *P*-value summary in Table 2. At 8 weeks of age, there was a significant (*P* < 0.001) reduction in vocalization rate with maze attempt, namely a 12% reduction from the first to second attempt followed by a negligible decrease (1%) between the second and third maze attempt. At both 27 and 41 weeks of age, no significant changes were seen in vocalization rate over three maze attempts.

Treatment significantly (*P* < 0.001) affected vocalization rate at 27 weeks of age, and this effect was dependent on maze attempt (*P* < 0.05). These statistical differences reflected the fact that vocalization rate was always highest in the GnRHa rams and on average increased (+14%), whereas it was always lowest and decreased (-37%) in the GnRHa+T group, and remained relatively constant in the Controls, over the three maze attempts. At 41 weeks of age, significant (*P* < 0.001) effects of treatment were again apparent, the GnRHa rams vocalizing the most and the GnRHa+T rams the least, regardless of maze attempt.

At 8 weeks of age, escape attempt rate decreased significantly (*P* < 0.001) with maze attempts (total of 211 escape attempts), regardless of treatment group. At 27 and 41 weeks of age, there were no effects of treatment, maze attempts or interaction between these factors on escape attempt rate (27 weeks of age: total of 33 attempts; 41 weeks of age: total of 36 attempts).

### 3.3 Long-term spatial memory

#### 3.3.1 Maze training

Treatment significantly (*P* = 0.020) affected the number of training attempts required to learn how to complete the maze within 1 min. The GnRHa+T group required fewer training attempts (3.5 ± 0.41) than the GnRHa group (6.2 ± 1.09), but neither of these two groups were different from the Controls (4.7 ± 0.67).

#### 3.3.2 Traverse times

**Fig. 5:**
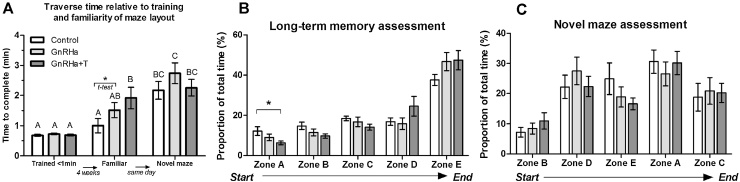
Summary of spatial performance at 45 weeks of age for long-term spatial memory and novel maze assessments. A: Traverse times at the end of training (41 weeks of age ‘Trained < 1min’), during long-term spatial memory assessment (‘Long-term memory’ using maze layout 1), and in an unfamiliar maze design (‘Novel maze’ using maze layout 2). Different letters on top of bars indicate significant differences between treatment group means, as determined by Tukey post hoc tests. B: Progression through maze zones during long-term spatial memory assessment. *P < 0.05 from Tukey post hoc test, when comparing the effects of treatment in that particular zone with a one-way ANOVA. C: Progression through maze zones during novel maze assessment. GnRHa: GnRHa-treated rams; GnRHa+T: GnRHa-treated rams that also received testosterone replacement. Control: untreated rams.

In Fig. 5A, the average traverse times are shown for the end of training, as well as when long-term memory was assessed, and when the maze traps were presented to animals in a novel order/orientation (‘Trained < 1min’, ‘Long-term memory’, ‘Novel maze’, respectively). All three groups had similar traverse times (*P* = 0.51) at the end of training. When comparing traverse times during long-term memory assessment with those at the end of training, traverse times were significantly (Time *P* < 0.001) longer for the GnRHa and GnRHa+T groups. There was also a significant (P = 0.032) effect of treatment, whereby the size of the effect tended to be different between the two treatment groups (Treatment × Time *P* = 0.085). Compared to the Controls, the GnRHa and GnRHa + T rams took 1.5 fold (TukeyHSD adjusted *P* = 0.090) and 1.9 fold (TukeyHSD adjusted *P* = 0.019) longer to complete the maze, respectively (One-way ANOVA of long-term memory assessment only: *P* = 0.043). Comparing traverse time during long-term memory assessment of only the Control and GnRHa groups (*t*-test), confirmed that the GnRHa group took significantly (*P* = 0.030) longer than the Control group.

During novel maze assessment (Fig. 1, Layout 2), traverse times for Control and GnRHa groups were significantly (*P* *<* 0.001) longer than in the familiar maze layout (Fig. 1, Layout 1). Treatment significantly (*P* = 0.015) affected novel maze traverse times, relative to long-term memory assessment, with a 1.2, 1.8 and 2.2-fold increase in the GnRHa + T, GnRHa and Controls groups, respectively (Treatment × Time *P* = 0.231). However, traverse times during the novel maze assessment were not different between treatment groups (One-way ANOVA of novel maze assessment only: *P* = 0.435).

#### 3.3.3 Progress through maze zones

The proportions of time animals spent in each maze zone, when long-term memory and the novel maze were examined, are shown in Fig. 5B & C. Overall, the proportionate time animals spent in each maze zone, when long-term memory was assessed, was similar except for zone E, in which they spent the most time. There was a significant (*P* < 0.05) effect of treatment on the proportion of time spent in zone A, where Controls spent the greatest, and the GnRHa+T group the least, proportion of time. A similar trend in the effects of treatment on proportionate time spent in a zone was seen in zones B and C.

When rams were tested in the novel maze layout, later the same day, the proportional time allocation pattern changed substantially, relative to that of the familiar maze (Fig. 5A, Long-term memory assessment). In the novel maze, animals passed relatively quickly through the first trap (zone B), but slowed down and spent approximately equal proportions of time in the second (zone D), third (zone E), and final (zone C) traps. The greatest proportion of time was spent in the fourth trap (zone A). Relative to the long-term memory assessment, the proportion of time spent in zone A was significantly (*P* < 0.001) increased, and zone E was significantly (*P* < 0.001) reduced during the novel maze assessment. There were no statistically significant effects of treatment on the proportion of time spent in any of the zones during the novel maze assessment, compared to the long-term memory assessment.

## 4 Discussion

This study demonstrated that there is an improvement, for male sheep from the early adolescent to young adult age range, in spatial orientation and learning performance, as well as behavioral measures of emotional reactivity that are displayed when performing spatial tasks. While the results do not support a significant role for GnRHa or testosterone on the outcome (i.e. traverse time) of spatial orientation and learning performance, they revealed that blockade of GnRH and testosterone signaling systems affected the manner in which animals moved through a spatial maze. Specifically, blockade of GnRH signaling increased, whereas restoration of testosterone signaling decreased, how quickly sheep progressed beyond a specific point in the maze and how emotionally reactive they were, i.e. how likely they were to vocalize or attempt to escape. Assessment of long-term spatial memory demonstrated that the blockade of GnRH signaling impaired spatial reference memory, and this effect was independent of testosterone replacement. In contrast, spatial working memory was primarily affected by the suppression of gonadal steroid signaling associated with the blockade of GnRH signaling.

### 4.1 Spatial orientation and learning

The findings, in the present study, that the blockade of GnRH signaling does not affect maze traverse times of male sheep during spatial orientation and learning assessments, are in agreement with the previous findings of Wojniusz et al. (2013). Minimum traverse times increased from 5 to 16 s in the current study’s modified 5-trap maze compared to the previous study’s 3-trap maze. These findings confirmed that the lack of a GnRHa-treatment effect on spatial orientation reported by Wojniusz et al. (2013) was not due to a ceiling effect associated with fast traverse times in a simple maze. Instead, the present study revealed that the manner in which sheep moved through the maze was affected by the blockade of GnRH and testosterone signaling, as concluded from the more detailed analysis of emotional reactivity and progression within the maze, together with the inclusion of an additional GnRHa-treated group in which testosterone was replaced. Specifically, it was seen that rams that had both GnRH and testosterone signaling blocked (GnRHa group) exhibited increased emotionally reactive behavior, as reflected by higher vocalization frequencies at 27 and 41 weeks of age (Fig. 4). This effect of peripubertal GnRHa-treatment was lost when testosterone signaling was replaced, which indicates that the effects were due to suppression of testosterone, secondary to the blockade of GnRHa signaling. Testosterone replacement also decreased the frequency of escape attempts as rams aged, independent of GnRHa-treatment (Fig. 4, Attempt 1), which again suggests that testosterone reduced emotional reactivity. This effect of testosterone has previously been reported in other species, typically in relation to reduction in fearful behavior (Boissy and Bouissou, 1994, Frye and Seliga, 2001, Aikey et al., 2002) or increase in hypothalamic-pituitary-adrenal axis responses to stress (Viau and Meaney, 1996, Seale et al., 2004).

The lower emotional reactivity of GnRHa+T animals may have indicated that this group was less anxious and motivated to traverse the maze to reunite with their peers, as reflected by their slower progression through the maze over multiple maze attempts at 41 weeks of age (Fig. 3), compared to Control and GnRHa groups. Differences in emotional reactivity may also explain the trend for GnRHa-treated animals to have faster traverse times, compared to GnRHa+T animals during spatial learning assessment (Fig. 2, Attempt 1 & 2 at 41 weeks of age), as they may have been more anxious and motivated to reunite with flock members. It is noteworthy that restorative effects of testosterone on spatial performance have been reported previously in adult male rats where it was shown to minimize the influence of non-mnemonic factors in a hippocampus-dependent version of the Y-maze (Hawley et al., 2013). That study is of particular note, as it also utilized an innate behavior − i.e. novelty-seeking of rats, whereas the current study used flocking in sheep − to study the effects of testosterone on spatial ability. It was argued that results from studies that used food reward or water escape as motivation to complete tasks, were compromised by the effects of testosterone (and other androgens) on food intake, body weight gain, anxiety and hypothalamic-pituitary-adrenal axis reactivity.

Suppression of gonadal steroid signaling is therefore likely to increase fearful behavior or elevate stress responses via the hypothalamic-pituitary-adrenal axis. Indeed, previous work (Evans et al., 2012) has indicated that the GnRHa-mediated blockade of pubertal hormone production increased anxiety and emotional reactivity in rams during a stress test. Thus, the loss of gonadal steroid signaling may alter the manner in which subjects behave during a spatial task, without affecting the performance outcome for spatial orientation and learning (i.e. no change in traverse time).

### 4.2 Long-term spatial memory

The successful retention and recollection of spatial information was reflected by Control rams retaining the ability to traverse the maze within 1 min, 4 weeks after training. This agrees with previous findings (Lee et al., 2006) that sheep are able to recall spatial information 6 weeks after training. Further evidence that animals retained spatial information was reflected in the pattern of maze progression during the long-term memory assessment. Familiarity with the maze layout was reflected by the fact that rams passed quickly through the first maze zone but spent a longer proportion of time in the last zone. This pattern of maze zone progression also became more prominent across the three attempts at 8 weeks of age and to a lesser extent across the three ages assessed, as animals became more familiar with the maze. Furthermore, this pattern of maze progression was lost when animals encountered an unfamiliar maze layout at 45 weeks of age. Finally, the similarity in the pattern of maze progression for the three groups would suggest that they all retained spatial information, even though traverse times indicated differences in long-term spatial recall.

A specific effect of GnRH signaling on long-term spatial memory was evident from the impaired ability of GnRHa treated animals to recall spatial information and that this effect could not be counteracted with the restoration of testosterone signaling. The lack of a restorative effect of testosterone on long-term spatial memory is interesting, because the GnRHa+T rams were the easiest to train to complete the maze within 1 min, but were the slowest to traverse the maze during the long-term memory assessment and exhibited slow maze progression during the assessment of spatial learning. However, it should be noted that the conditions during training for the assessment of long-term spatial memory differed from those of the spatial learning assessment. Specifically, maze attempts during long-term memory training followed immediately after one another and animals were shown how to complete the maze after 1 min, whereas spatial learning assessment constituted three attempts that were separated by approximately 2 h and rams that did not complete the maze within 5 min were guided back to the audience pen via the entrance. Thus the apparently conflicting results could reflect differences in the learning pattern of the GnRHa+T group across these two components of the study, i.e. effects on rapid repetitive learning vs. the longer timescale used in the spatial learning assessment. Furthermore, it could reflect the effects of testosterone in decreasing emotional reactivity and motivation to complete the maze, as discussed above. We can, therefore, not entirely rule out the possibility that the effects of long-term peripubertal GnRHa-treatment on spatial memory were not, in part, mediated via differences in motivation or emotional reactivity in this study.

Comparison of performance in long-term memory and novel maze assessments is of interest as it provides a means to assess whether blockade of GnRH/testosterone signaling had an effect on spatial reference or spatial working memory. Spatial reference memory is defined as the categorization of information that remains the same among trials, which is comparable to having the same traps in multiple assessments (Olton and Papas, 1979). Spatial working memory refers to the categorization of information about a particular sequence of spatial cues, which is comparable to changing the order of traps in the novel maze assessment (Olton and Papas, 1979). Performance during long-term spatial memory and novel maze assessments was similar for both groups in which GnRH signaling was blocked (GnRHa & GnRHa+T), irrespective of testosterone replacement. However, testosterone reversed the increase in traverse times from familiar to novel layouts when GnRH signaling was blocked (GnRHa group). Therefore, it is concluded that spatial working memory was influenced by testosterone signaling, whereas spatial reference memory was explicitly influenced by GnRH signaling. This conclusion is supported by work in adult male rats, which also noted that spatial working memory was more sensitive to testosterone than spatial reference memory (Sandstrom et al., 2006, Spritzer et al., 2008; Hawley et al., 2013).

### 4.3 Potential mechanisms involved

While endogenous GnRH secretion is pulsatile, the continuous GnRHa administration in this study suppressed signaling downstream of the GnRH receptor. The exact changes induced by GnRHa-treatment within the central nervous system are likely to be complex and involve an array of altered pathways. Here we discuss the potential mechanisms involved with the suppression of GnRH and testosterone signaling, from evidence in the literature, without having direct measures of GnRH or testosterone in circulation, cerebrospinal fluid, or tissue homogenates in this study.

The hippocampus is central to spatial learning and memory, and Albertson et al. (2009) has demonstrated that there are GnRHR type I immunoreactive cells in the CA1 to 4 regions, as well as the dentate gyrus, of the ovine hippocampus. Nuruddin et al. (2013b) reported that peripubertal blockade with GnRHa, resulted in sex specific changes in hippocampal (CA1 to 3) mRNA expression. Specifically, while there were no changes in the levels of expression of mRNA for GnRH receptor (Types I and II) and estrogen receptor (alpha and beta), in rams, mRNA expression of endocrine variables (androgen receptor, aromatase and growth hormone), as well as neuroplasticity markers (neural cell adhesion molecule 1, VGF nerve growth factor inducible and LIM homeobox 5), decreased and mRNA expression for the AMPA1 glutamate receptor, an additional neuroplasticity marker, increased, relative to the Controls. Alterations in LIM homeobox 5 are associated with hippocampal development, where it is vital in regulation of precursor cell proliferation, as well as the control of neuronal differentiation and migration (Zhao et al., 1999). The AMPA1 glutamate receptor plays an important role in long-term potentiation, which is − together with its counterpart long-term depression − an activity-dependent synaptic plasticity mechanism involved in learning and memory (Benke et al., 1998). An increase in the number of post-synaptic AMPA1 glutamate receptors, their probability to be open, their kinetics or an increase in their single-channel conductance would generally indicate improved learning and memory (Benke et al., 1998). In the present study, GnRHa-treated animals showed impaired spatial learning and memory, despite the increase in AMPA1 glutamate receptor mRNA that was reported by Nuruddin et al. (2013b) using the same ovine model. However, mRNA levels do not necessarily reflect protein expression, or any other alterations in AMPA1 glutamate receptor function, and would be valuable to study these in the present cohort of animals.

Androgen receptors mediate alterations in synaptic plasticity in the hippocampus (MacLusky et al., 2006) and it has been suggested that impaired spatial memory in adult rats, following gonadectomy, occurs due to reduced expression of hippocampal androgen receptors (particularly in area CA1) (Hawley et al., 2013). Such a reduction in mRNA expression of androgen receptor and aromatase reported by Nuruddin et al. (2013b) in GnRHa-treated rams, provides support that long-term GnRHa-treatment may alter the way in which subjects respond to testosterone. Ward et al. (1999) also reported that altered receptor expression in the brain, rather than altered gonadal steroid production, was responsible for underperformance in copulatory behavior that resulted from prenatal exposure to stress and alcohol. Furthermore, the study by Seale et al. (2004) provided evidence that restorative effects of testosterone replacement on hypothalamic-pituitary-adrenal axis stress responses, in gonadectomized male rats, are mediated via the androgen receptor. Peripubertal GnRHa-mediated differences in hippocampal androgen receptor mRNA may therefore also relate to the observed differences in emotional reactivity in the present study. However, further physiological evidence of steroid receptor expression and circulating steroid levels is required to investigate what effects would be associated with GnRHa-treatment and testosterone replacement and how this relates to the differences observed in spatial memory.

Finally, the suppression of LH signaling that is associated with GnRHa-treatment, may also play a role in the observed changes in learning and memory, as LH receptors are expressed in the CNS, including the hippocampus (Blair et al., 2015). In addition, it has been shown in human and rodent studies that the age-related increase in circulating LH and decline in gonadal steroid production, correlates with impaired cognitive function (Blair et al., 2015). However, the route through which circulating LH could reach receptors in the central nervous system is not yet characterized.

## 5 Conclusion

The results of this study demonstrate that spatial orientation and learning were indirectly affected by peripubertal GnRHa-treatment, whereby the blockade of testosterone signaling, rather than the blockade of GnRH signaling only, increased emotional reactivity and motivation to reunite with flock members. Long-term retention of spatial information was impaired by the specific blockade of GnRH signaling, and did not improve when testosterone signaling was restored. It is therefore concluded that blockage of the pubertal transition with GnRHa-therapy greatly affected long-term spatial memory, particularly with regard to spatial reference memory, but minimally affected spatial orientation and learning in males. Furthermore, the restoration of testosterone signaling exaggerated changes in spatial reference memory and counteracted the changes in spatial orientation and spatial working memory. It is not known whether peripubertal GnRHa-treatment in females will also result in a reduction in long-term spatial memory. Further investigation is required to establish the mechanisms through which GnRHa alter spatial memory and learning − whether it is directly via GnRHR in the hippocampus or indirectly via changes in emotional reactivity − and if these effects are reversible when GnRHa-treatment is terminated after the normal post-pubertal age.

## Figures and Tables

**Table 1:** Spatial Orientation. Summary of two-way ANOVA *P*-values to assess the effects of treatment on performance during the first maze attempt at ages 8, 27 and 41 weeks of age.

Response Variable	Treatment	Age	Treatment × Age
Traverse time	0.356	**<0.001**	0.754

Proportion of time			
*Zone A*	0.648	**<0.001**	0.902
*Zone B*	0.713	**0.013**	0.583
*Zone C*	0.602	0.371	*0.081*
*Zone D*	0.737	**<0.001**	0.895
*Zone E*	0.697	**<0.001**	0.866

Emotional reactivity			
*Vocalization rate*	0.148	**<0.001**	0.783
*Escape attempts*	0.373	**<0.001**	**0.014**

**Table 2:** Spatial learning. Summary of two-way ANOVA *P*-values to assess the effects of treatment on performance across all three maze attempts within the same day at ages 8, 27 and 41 weeks.

Response Variable	8 wks	27 wks			41 wks		
	Attempt	Treatment	Attempt	Treatment × Attempt	Treatment	Attempt	Treatment × Attempt
Traverse time	**<0.001**	0.281	**<0.001**	0.391	0.164	**<0.001**	0.807

Proportion of time							
*Zone A*	**<0.001**	*0.099*	**<0.001**	0.786	0.715	*0.094*	0.498
*Zone B*	0.466	0.678	**0.004**	0.423	0.672	**0.007**	0.859
*Zone C*	0.657	0.312	**0.045**	0.490	0.342	**<0.001**	0.267
*Zone D*	0.205	0.856	0.354	0.428	**0.025**	**<0.001**	0.944
*Zone E*	**0.028**	0.331	0.936	0.879	0.224	0.506	0.749

Emotional reactivity							
*Vocalization rate*	**<0.001**	**<0.001**	0.802	**0.019**	**<0.001**	0.966	0.909
*Escape attempts*	**<0.001**	0.316	0.378	0.497	0.646	0.391	0.326
